# A Simplified Microscopy Technique to Rapidly Characterize Individual Fiber Traits in Cotton

**DOI:** 10.3390/mps6050092

**Published:** 2023-10-03

**Authors:** Quinn LaFave, Shalini P. Etukuri, Chaney L. Courtney, Neha Kothari, Trevor W. Rife, Christopher A. Saski

**Affiliations:** 1 Department of Plant and Environmental Sciences, Clemson University, Clemson, SC 29634, USA; qlafave@g.clemson.edu (Q.L.); setukur@g.clemson.edu (S.P.E.); ccourtn@clemson.edu (C.L.C.); 2 Cotton Incorporated, Cary, NC 27513, USA; nkothari@cottoninc.com

**Keywords:** phenotyping, cotton, measurements, fiber, microscopy

## Abstract

Recent advances in phenotyping techniques have substantially improved the ability to mitigate type-II errors typically associated with high variance in phenotyping data sets. In particular, the implementation of automated techniques such as the High-Volume Instrument (HVI) and the Advanced Fiber Information System (AFIS) have significantly enhanced the reproducibility and standardization of various fiber quality measurements in cotton. However, micronaire is not a direct measure of either maturity or fineness, lending to limitations. AFIS only provides a calculated form of fiber diameter, not a direct measure, justifying the need for a visual-based reference method. Obtaining direct measurements of individual fibers through cross-sectional analysis and electron microscopy is a widely accepted standard but is time-consuming and requires the use of hazardous chemicals and specialized equipment. In this study, we present a simplified fiber histology and image acquisition technique that is both rapid and reproducible. We also introduce an automated image analysis program that utilizes machine learning to differentiate good fibers from bad and to subsequently collect critical phenotypic measurements. These methods have the potential to improve the efficiency of cotton fiber phenotyping, allowing for greater precision in unravelling the genetic architecture of critical traits such as fiber diameter, shape, areas of the secondary cell wall/lumen, and others, ultimately leading to larger genetic gains in fiber quality and improvements in cotton.

## 1. Introduction

Cotton is a preeminent natural fiber crop that is extensively cultivated in nearly 80 countries worldwide for its natural fiber [1]. In addition to its lint, the cottonseed is also a valuable component of the plant and serves as a vital source of oil and protein [2]. The lint, which is the cotton fiber, is primarily used in the textile industry for clothing. Linters, known as short fuzz on the seed, provide cellulose for making explosives, plastics, and many other products [3]. The cottonseed, on the other hand, offers oil, meal, and hulls [3]. The global annual consumption of cotton fiber is approximately 27 million metric tons, or approximately 115 million bales [4]. Cotton breeders strive to improve fiber quality and yield to provide a better alternative to synthetic fibers derived from petroleum, which has significant environmental impacts [5]. Researchers are also exploring methods to modify cottonseed for human consumption and livestock feed [6].

In the United States, cotton production primarily comes from *Gossypium hirsutum* (Upland cotton) and *Gossypium barbadense* (Pima cotton), which account for 16.1 million bales and 400 thousand bales, respectively [7]. The widespread cultivation of Upland cotton can be attributed to its better adaptability to harsh environmental conditions, high production, and greater yield potential [8]. Conversely, Pima cotton is known for its superior fiber quality attributes such as length, strength, and fineness. Pima cotton is distinct from Upland cotton in various traits, including growing habits, adaptations, and yield [9]. Attempts to introgress genes for elite Pima fiber traits into high-yielding commercial Upland lines have been limited due to the incompatibility of their genomes [10].

Cotton fibers arise as single-celled structures emerging from epidermal cells of ovules [11]. The differentiation and development process encompasses four overlapping stages: initiation, elongation involving primary cell wall biogenesis, Secondary Cell Wall (SCW) biosynthesis, and fiber maturation [12,13]. Elongation involves the accumulation of crystalline cellulose within the secondary cell wall, forming the lumen wall that encloses an internal space called the lumen. The lumen, an essential conduit for nutrient transfer during growth [14], is characterized by a hollow canal. Initially, during the growth phase, the fiber exhibits an approximately circular cross-section. However, as the fiber undergoes desiccation, its shape becomes irregular, assuming a kidney-shaped appearance that is contingent upon the thickness of the secondary cell wall [15]. Specific genetic mechanisms regulate key fiber attributes such as length, strength, uniformity, and micronaire values [16]. Micronaire measures the air permeability of a consistent mass of compressed cotton fibers within a fixed volume [17]. Among these attributes, fiber strength and length hold paramount importance for yarn quality [18], whereas micronaire value impacts dyeing consistency and processing (spinning). It is worth noting that the micronaire value serves as an indicator of both fiber maturity and fineness [19,20,21]. However, it should be emphasized that micronaire readings represent a relative scale and cannot be solely relied upon to assess either fineness or maturity. Higher micronaire values are associated with more mature and coarser fibers, whereas lower micronaire values indicate finer and less mature fibers [20,21].

Assessing cotton fiber quality is a complex process that requires precise and accurate measurement of various fiber quality traits [22]. For over two decades, the High-Volume Instrument (HVI) has been the primary source of measurement for fiber quality and selection improvement in the cotton industry. However, the HVI system has limitations in evaluating the contribution of fiber length variations within a sample [23]. The Advanced Fiber Information System (AFIS) has emerged as a complement to the HVI for fiber quality measurement, especially fiber length distribution within a sample, providing more accurate single-fiber quality information [24]. Several studies have demonstrated that the AFIS is an effective tool for evaluating yarn quality and spinning performance, surpassing the HVI in terms of measuring mean fiber values and distributions and precisely estimating fineness and maturity through cross-sectional analysis [23]. Nevertheless, further research is needed to fully understand the potential for the AFIS to provide more helpful information regarding fiber length distribution and how it can be utilized in the cotton industry.

Maturity and fineness are two critical parameters used to evaluate cotton fiber quality. Highly mature fibers produce good-quality yarns and have good dye affinity. However, despite the significance of these fiber characteristics, no measurement methods are satisfactory, quick, and reliable [25]. The lack of reference standards for maturity has made it impossible to validate the existing instruments, such as double compression airflow instruments and the maturity module in the AFIS [24]. There are many indirect methods for measuring cotton fiber maturity; however, direct determinations can be performed only by microscopic processes, which is the definitional reference for measuring maturity and fineness [26]. In 1956, Lord developed 100 cottons to validate micronaire [25]. Gravimetric fineness was determined by the “cut and weigh” method. However, this method introduces bias into the measurements, as the sample taken is not independent of the fiber length [24]. In the 1980s, the International Textile Manufacturers Federation (ITMF), in collaboration with other organizations, established a set of nine calibration cottons [26]. In 1999, Thibodeaux and Rajasekaran [27] used a commercial image analysis system to measure the cross-sectional images of ~50 cotton varieties showing a wide range of genetic fineness. Despite these significant efforts, none of these methods have achieved widespread adoption today. The Commonwealth Science and Industrial Research Organization (CSIRO) developed the Cottonscan instrument [28,29,30,31,32] for the direct measurement of fiber fineness and the Siromat instrument for measuring fiber maturity distribution using polarized light microscopy [33,34,35,36]. In 2018, ITMF recognized the commercialization of Cottonscan/Siromat. This instrument measures the diameter of thousands of cotton fiber snippets in just 30 s and estimates the fiber maturity ratio using two procedures. Although it produces results within a few seconds without the need for resin embedding, it is worth noting that the sodium hydroxide solution used in procedure 1 is caustic and corrosive. This method does not provide direct measurements, unlike the approach presented in this paper [28,29].

Cross-sectional analysis offers direct and precise measurements of fiber maturity and fineness, serving as reference data to calibrate or validate other indirect measurements of these fiber characteristics. However, in spite of the significance, cross-sectional measurements have not been widely applied to cotton fiber quality evaluations due to tedious procedures for both sample preparation and image analysis [30]. The technique of directly measuring fiber cross-sections under an optical microscope, known as first-principles analysis, poses significant technical challenges that demand a skilled operator. Additionally, this method is labor-intensive and time-consuming. As a result, until recently, there was a lack of a universally accepted reference cotton data set with assigned values for fiber maturity and fineness [31]. Hequet et al. addressed this challenge with an extensive and detailed study aimed to create a set of 104 reference cottons for fiber maturity measurements [24]. In their study, they used a modified version of the methacrylate embedding method for fiber embedding and cross-sections developed by Boylston at the United States Department of Agriculture Southern Regional Research Center (USDA-SRRC) in New Orleans, Louisiana, USA [32,33] and the Fiber Image Analysis Software (FIAS) image analysis system developed by Xu and colleagues [30,34,35]. Although this method is more accurate, it is tedious, requires harmful chemicals, and cannot be used to rapidly assess many samples.

This study presents a novel approach for simplified and efficient fiber histology, cross-sectioning, and image analysis of individual fibers. The method involves detailed analysis of latitudinal fiber cross-sections acquired through a combination of super-resolution light microscopy and machine learning techniques. This approach establishes a first-generation analytical tool capable of automatic detection of viable fibers in an image, enabling accurate determination of key measurements while reducing experimental error. The obtained measurements provide precise quantitative data on fiber properties, specifically (1) fiber circumference and area, (2) lumen circumference and area, and others.

## 2. Experimental Design

The primary objective of this study was to establish a standardized protocol for obtaining consistent and high-quality measurements of individual cotton fibers, with validation conducted using a subset of the reference cotton material generated by Hequet et al. [24]. To accomplish this, we devised an embedding technique where mature cotton fibers were embedded within a resin medium that preserves the natural sample structure. This approach facilitated efficient embedding of a large number of fibers within a single section while enabling individual fiber analysis. Given the inherent variability in cotton fiber sizes, even within the same boll, a substantial number of fibers were analyzed to ensure precise estimation of fiber properties. Key morphological characteristics of interest such as the diameter and area of the crystalline structure, as well as the lumen, were measured. To evaluate critical fiber traits, ten distinct cotton lines were examined, and subsequent statistical analysis was used to compare fiber properties within these lines. Notably, the experiment was conducted under standard laboratory conditions, with temperatures maintained within the range of 20 °C to 25 °C, and humidity levels between 30% and 50%.

### 2.1. Materials

1. Hemostat (Surgicalonline, Dix Hills, NY, USA; Amazon; B07CRTRJFY)
2. Fine-Toothed Comb (Leinuosen, China; Amazon; B07Q45NB93)
3. 24′ Galvanized Wire (The Hillman Group, Cincinnati, OH, USA; Cat.no.: 123132)
4. Size 00 Gelatin Capsules (Capsuline, Fort Lauderdale, FL, USA; X00RQSE8X)
5. Teflon Tubing (2 mm inner diameter × 4 mm outer diameter, Amazon; 3DPTFE2/4CM5FT)
6. 5 mL Microfuge tubes (Axygen, Tewksbury, MA, USA; H108MCT-500-C37)
7. London Resin White (Electron Microscopy Sciences, Hatfield, PA, USA; Cat.no.: 14381-UC)
8. LR White Accelerator (Electron Microscopy Sciences; Cat.no.: 14385)
9. 3 mL Needle Syringes (Becton Dickinson & CO., Franklin Lakes, NJ, USA; Cat.no.: 003829003095742)
10. Positively Charged Microscope Slides (Tanner Scientific, Sarasota, FL, USA)

### 2.2. Equipment

- Vortex (Scientific Industries, Bohemia, NY, USA; G560)
- Microfuge (Heathrow Industries, Vernon Hills, IL, USA; 3079140)
- Glass Microtome (Leica Biosystems, Deer Park, IL, USA; Leica RM2265)
- Sputter Coater (Ladd Research Industries, Essex Junction, VT, USA; Hummer 6.2)
- Scanning Electron Microscope (Hitachi High Technologies America, Inc., Dallas, TX, USA; Hitachi S-3400N)
- Light Microscope (Evident Scientific.com, Waltham, MA, USA; Olympus LEXT Optical Profiler)

## 3. Procedure

### 3.1. Fiber Embedding

**Figure 1:**
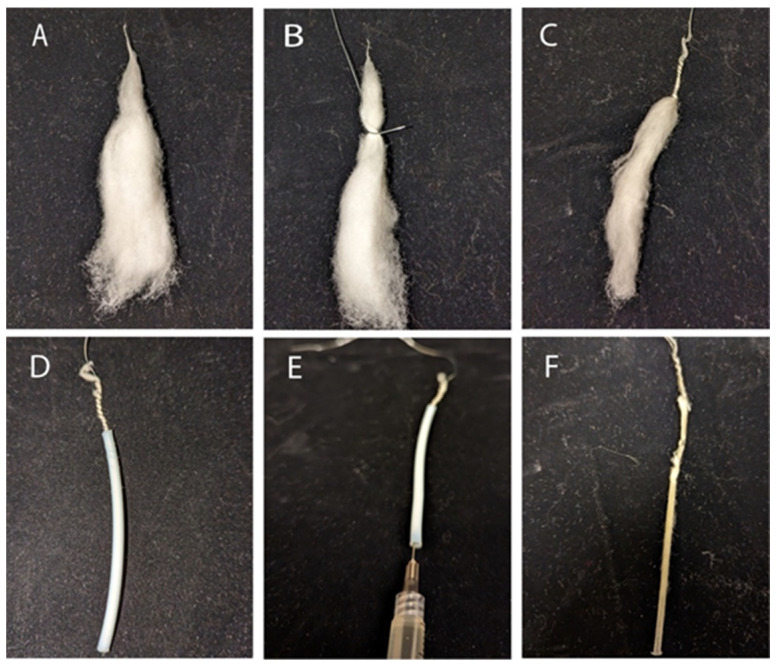
Cotton fiber preparation for embedding. (**A**) Cotton fiber bundle; (**B**,**C**) the cotton fiber bundle is wrapped with galvanized wire; (**D**) cotton fiber bundle in Teflon tubing; (**E**) injecting the polymerization solution; (**F**) polymerized cotton fiber bundle.

**Figure 2:**
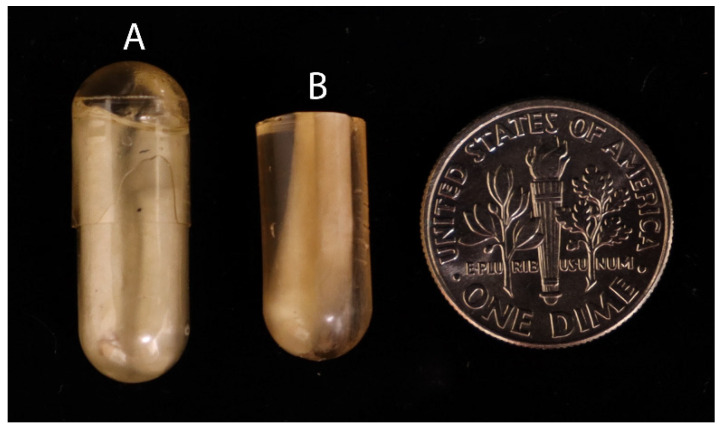
Embedded capsule preparation. (**A**) Polymerized resin with embedded fiber in capsule; (**B**) cut capsule section of embedded fiber. (US dime for scale.)

1. Obtain 0.1 g of matured dried cotton fiber, remove seeds and debris, comb 2 inches with a fine-toothed comb, and twist the tip to secure the bundle (Figure 1A).
2. Loop 24-gauge galvanized wire around 3 inches of the bundle, leaving excess wire facing away from it. Tightly wrap ~1/3 of the bundle with the wire (Figure 1B,C).
3. Insert the free wire end through ~1.75 inches of Teflon tubing. Ensure that the wire and fiber slide freely through the tube together and comb the fiber to reduce density if there is resistance (Figure 1D).
4. Combine 1 mL of uncatalyzed London Resin (LR) White with 2.5 μL of accelerator in a 5 mL microfuge tube and mix using a vortex for 10 s. Inject the solution into the Teflon tube with a syringe. Allow the bundle to polymerize inside the tube for 5–10 min (Figure 1E).
5. Pull the bundle out of the tube by grasping the free wire end and pulling it straight or using a hemostat to grip and pull it out in a straight line. The resulting bundle should be solid and easy to cut (Figure 1F).

1. Obtain a size 00 gelatin capsule and cut the polymerized bundle into segments that fit inside the capsule, excluding any segments containing wire. Multiple segments can be inserted into the capsule.
2. Combine a new mixture of 1 mL of uncatalyzed LR and 2.5 μL of accelerator and vortex it for 10 s in a 5 mL microfuge tube. Add the mixture to the capsules and quickly cap and spin them using a microfuge for 10 s (Figure 2A).
3. Polymerization should take 5–10 min and should not release excessive heat. Remove the gel capsules using a single-edged razor by cutting between the gel capsule and resin (Figure 2B).

### 3.2. Sectioning

1. Trim away the uneven parts of the non-rounded end of the resin to create a flat end.
2. Prepare a Leica RM2265 glass microtome and obtain a fresh 9 mm glass microtome blade. Ensure that the glass blade’s width is the same or larger than the capsule width.
3. Cut out 6 μm sections from the resin. Move full sections into the water bath at 44 °C, clearing the working area of the microtome after each section, and remove incomplete sections.
4. Isolate the first section and examine it using a compound microscope at 100×power for fiber visibility. If you do not detect fibers, discard the section and create new sections until you verify the presence of fibers.
5. After verification, add more sections to the slide. Then, put the slide onto a slide warmer set to 37 °C and let the slides dry for 30 min to an hour.

**Figure 3:**
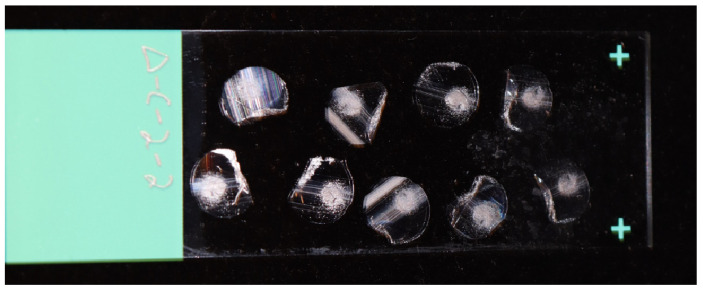
Cut fiber cross-sections on positively charged glass slide.

The quality of microtome sections is highly dependent on the condition of the glass blade used for sectioning. Any defects or nicks on the blade can cause a “dragging” appearance in the section, which is indicative of tearing rather than a clean cut. Therefore, to avoid issues with sectioning, it is recommended to use a fresh glass knife and to replace it regularly throughout the sectioning process. Figure 3 provides an example of well-cut sections, where most of the section is clear, with occasional lines being acceptable and not significantly affecting imaging or data acquisition. It is not uncommon for some sections to fold over themselves. The degree of polymerization of the resin is a crucial factor to consider, as over-polymerization can lead to the rapid dulling of the blade, as opposed to properly polymerized resin. The number of required fibers for imaging will determine the number of sections that can be added to a single microscope slide. After the drying process, some sections may loosen and fail to lie flat on the slide. In such instances, re-wetting the slide and allowing it to dry again can facilitate adhesion of the sections to the slide. To improve imaging outcomes, the use of positively charged slides is suggested, as they enhance section adhesion and facilitate flatness.

### 3.3. Microscopy

1. To perform SEM imaging, coat the microscope slide containing dried sections using a Ladd/Hummer 6.2 sputter coater. Obtain images using the Hitachi 3400 Scanning Electron Microscope at 1000× or 2000× magnification, ensuring to include a reference distance for subsequent analysis.
2. For light imaging, utilize the Olympus LEXT Optical Profiler. Capture three-dimensional light-based images of the sections within a range of 8–15 μm. Due to creases within the sections, the imaging speed is slightly slower. Image the sections at 100×, obtaining multiple images of a wide view. Subsequently, stitch these images together to create a larger image with high resolution.

**Figure 4:**
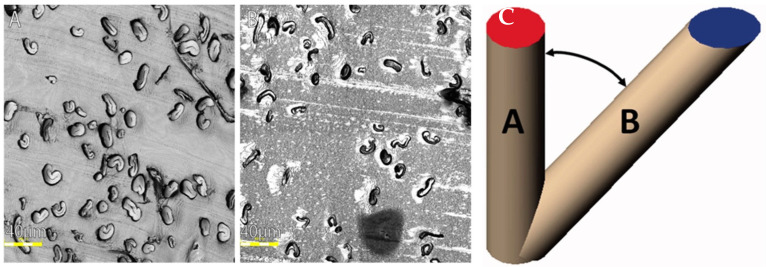
Acceptable vs. inadequate light microscope images. Imaged sections at 100× resolution. (**A**) The representative section of the slide showcases ideal embedded and sectioned fiber images. Although some fibers were embedded horizontally, the majority were embedded vertically. The actual embedding process followed the appropriate speed and temperature, considering that the fibers are mostly kidney-shaped and the lumens are not tightly closed; (**B**) the representative image illustrates poor-quality sectioning (irregularities in the resin) and misformed fibers resulting from improper embedding. Figure 4B demonstrates the consequence of using excess accelerator, leading to both improperly sectioned images and the heat and speed of polymerization causing the fibers to become misshapen. Right (**C**). Graphical depiction of error induced when fibers are not oriented to a vertical axis, taken from [15].

An example of contrasting section quality is shown in Figure 4A,B below. Additionally, the degree of polymerization of the resin is a crucial factor since over-polymerization can lead to the blade rapidly dulling. Over-polymerization also leads to excess heat release during the polymerization process. Excess heat can lead to morphological changes in the fiber that will not revert to the original shape due to the surrounding polymerization. The embedding technique is critical, as fibers should be as perfectly close to the fiber axis as possible for precision with measurements. Deviations from the normal fiber axis will influence the actual observable surface area as the plane changes, as shown in Figure 4C.

**Figure 5:**
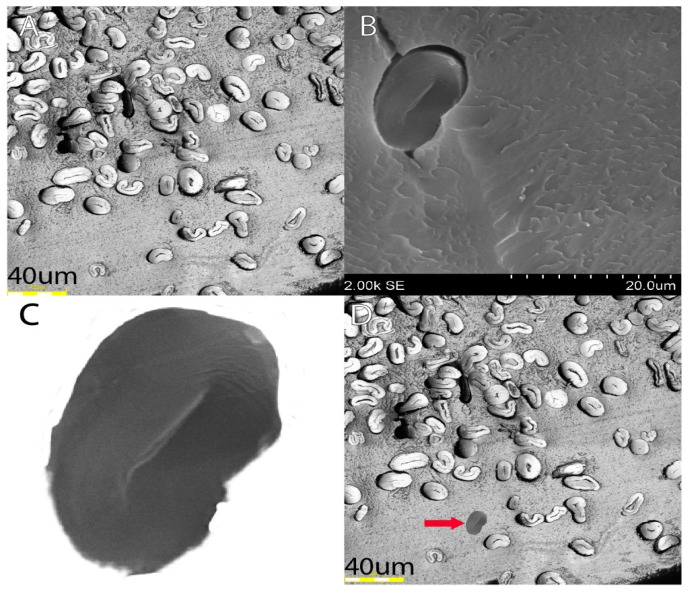
SEM fibers vs. light image fibers. (**A**) Shows a quality light image of fibers from a certain line; (**B**) shows an image of a singular fiber from the same fiber line; (**C**) shows the isolated fiber; (**D**) shows what the fiber would look like if inserted into the light image at relative size.

Despite being taken at different magnifications, no detectable difference (Table 1 and Figure 5D) occurred between measurements of fibers taken using SEM (Figure 5B) or light images (Figure 5A). When obtaining images, proper naming of the files will ease all subsequent processes. Images were classified by line, capsule #, slide # (which should only contain consecutive sections from the same capsule), and image #. Naming the images using this naming convention allowed for any discrepancies within the data that were caused by error to be easily identified.

### 3.4. Manual Fiber Image Analysis in Adobe Photoshop

**Figure 6:**
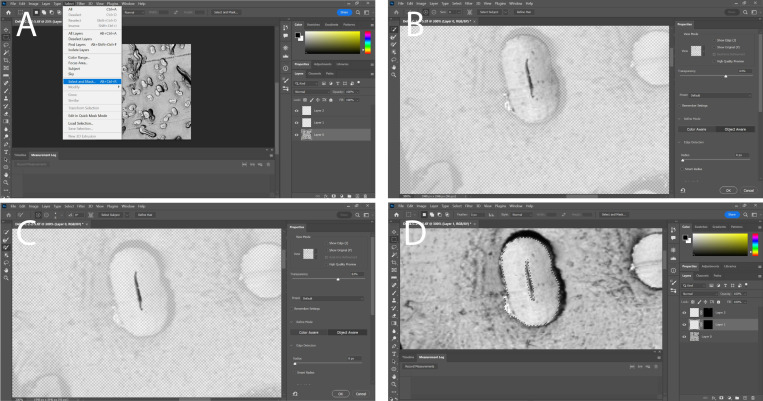
Manual image processing and measurements of fiber characteristics. (**A**) Selection of mask, note how the mask is being created on the original layer; (**B**) creation of a mask for the entire fiber itself, the smart tool brush is used as it can easily detect the edges of a fiber and accurately trace it; (**C**) creation of a mask for the lumen only covers a very small area of the actual fiber, the regular brush tool is used for this as it gives greater control over the mask; (**D**) the masks have been moved to different layers and have both been added to the selection showing an outline of the mask overlayed over the original image.

1. In Adobe Photoshop, open the fiber image and unlock its layer. Then, create two new layers on the image.
2. To create a mask on the original layer of the image, highlight both the upper surface of the crystalline structure and the lumen. Generate a new mask by selecting “Select” followed by “Select and Mask” (Figure 6A). Use one of the brush tools to highlight the fiber (Figure 6B) or the lumen (Figure 6C) entirely. This will generate a mask that represents what was highlighted.
3. Transfer the mask to a different layer by dragging and dropping it. Generate a new mask on the original layer to isolate the other measurement of the same fiber. Finally, add the new mask to its own layer (Figure 6D).
4. Repeat steps 4–6 for every fiber within the image.
5. Create separate fiber and lumen masks for all fibers within an image (Figure 5D). Utilize the ruler tool to measure the reference distance and document the measurement in pixels in the “measurement log” tab by clicking on “Record Measurement”. Generate a custom measurement scale in the measurement log by converting the reference distance value to the number of pixels used in the reference distance.
6. Create a new “Custom Data Points” section to include the following fields: “Document”, “Area”, “Perimeter”, “Circularity”, “Height”, and “Width”.
7. To select the mask for the lumen of one of the fibers, right-click on the mask and choose “Add mask to selection”. Then, click on “Record Measurement” to save the measurement.
8. Next, select and record the mask for the crystalline structure of the same fiber. Afterwards, deselect the mask.
9. Repeat steps 7–8 for each fiber within the image.
10. For each image of interest, repeat steps 2–9. After measuring all the images, select and export all the measurements to a notepad document. Edit the notepad document to ensure that both the lumen and fiber measurements are on the same row, and then copy these rows into an Excel document.

Because multiple fibers are present in each image, each fiber’s identity must be uniquely labeled. To achieve this, we assigned a letter to each fiber based on its relative position within the image, from left to right and top to bottom. Specifically, the naming convention involved adding a letter corresponding to each fiber’s position. For instance, in an image containing four fibers situated at each corner, the top-left fiber was designated as “A”, and the bottom-right fiber was identified as “D”. By utilizing this approach, we were able to accurately distinguish and label each fiber in the image.

**Figure 7:**
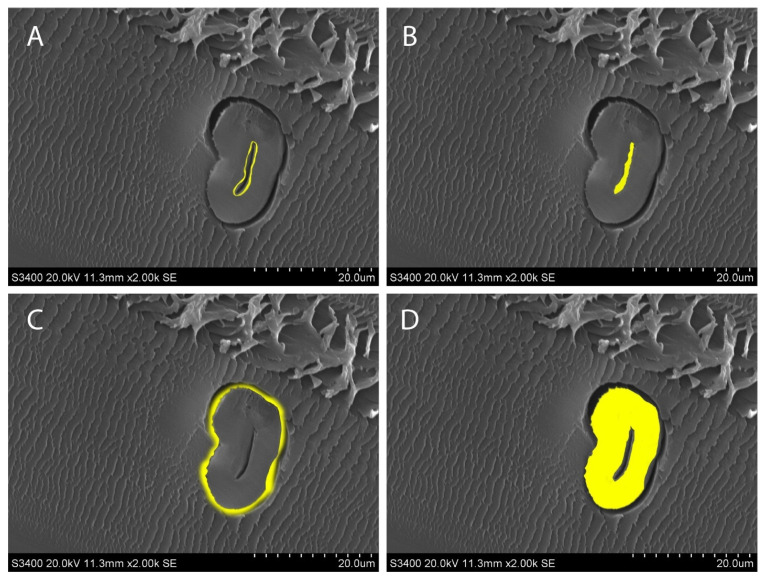
Measurements of interest shown in yellow. (**A**) The lumen circumference; (**B**) lumen area; (**C**) fiber circumference; (**D**) fiber area.

Figure 7 shows the measurements of interest in this experiment. The creation of a mask allows both measurements to be constantly edited and verified to ensure that only the areas of interest are measured.

### 3.5. Data Analysis

1. Verify that all values within the Excel file containing all fiber measurements are filled and properly converted from pixels to μm.
2. Create new columns for the fiber measurements “Outer True Area”, “Outer/Lumen”, and “Line”.
3. Calculate the “Outer True Area” by subtracting the value given for the area of the crystalline structure from the Lumen Area for the same fiber.
4. Divide the “Outer True Area” by the Lumen Area to obtain the “Outer/Lumen” ratio.
5. Recording the line from which the cotton sample is taken creates the “Line” category.
6. Next, create an Excel file with a minimum of 14 columns, which should include “Name”, “Lumen Area”, “Lumen Perimeter”, “Lumen Circularity”, “Lumen Height”, “Lumen Width”, “Outer Area”, “Outer True Area”, “Outer Perimeter”, “Outer Circularity”, “Outer Height”, “Outer Width”, “Outer/Lumen”, and “Line”.
7. Subsequently, analyze the data set using the JMP software.
8. Under the “Analyze” tab, select the “Fit X by Y” option.
9. For the “Y, Response”, use all columns except for “Name” and “Line”.
10. Set the “X, Factor” as “Line”.
11. Next, determine the Quantiles, Means, and Standard Deviations.
12. Conduct an ANOVA test on each of the lines.
13. Additionally, perform an all-pairs Tukey–Kramer HSD test and conduct a Student’s test for each pair to detect differences between each of the lines for every parameter.

### 3.6. Comparison to Paraffin Embedding and Resin Embedding

**Figure 8:**
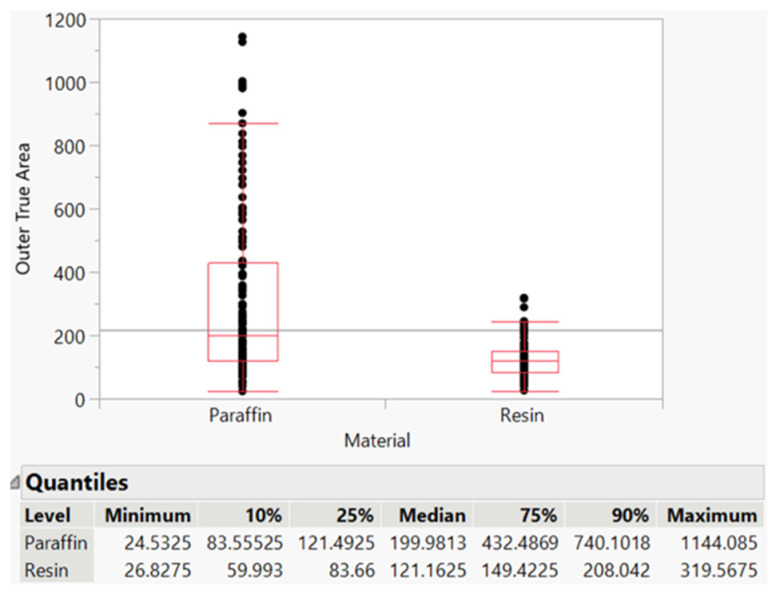
Paraffin vs. resin fiber area distribution.

As previously stated, there was a switch from paraffin to resin, as it allowed for easier, more efficient, and more controlled isolation of cotton fibers. Figure 8 shows the distribution differences between paraffin and resin, with paraffin having a larger range of values (see the maximum of paraffin vs. maximum of resin), which could be attributed to error introduced by the embedding method.

**Figure 9:**
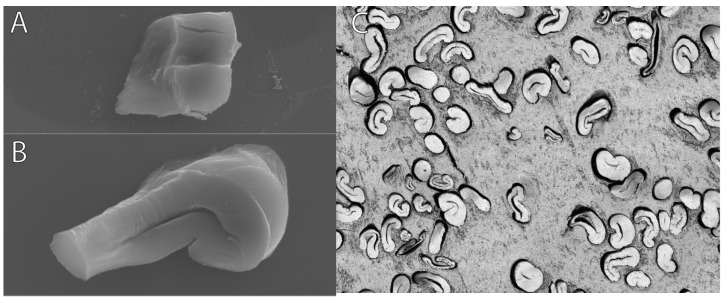
(**A**,**B**) Paraffin-embedded and SEM fibers; (**C**) London Resin White and light microscope fibers. These images depict the differences in the quality of the fibers from both methods.

The objective of the embedding technique was to discover a means of efficiently embedding a substantial quantity of cotton fibers while producing accurate representations of these fibers in images, all without causing any damage or alteration to the fiber morphology. The way the fibers were embedded in paraffin required chemicals for the deparaffinization process, which could flip over the fibers (Figure 9A). Coating the fibers in paraffin also did not allow for fibers to be properly sectioned via microtome (Figure 9B). While fibers embedded in LR White resin were embedded correctly, imaging under the light microscope provided more individual cross-sections per image and are suitable for analysis compared to paraffin-embedded and SEM fibers (Figure 9C).

### 3.7. Automated Image Analysis and Characterization of Fibers

To further increase the rate and accuracy of cotton fiber phenotyping, state-of-the-art deep learning models were trained to regionalize and measure individual fiber cross-sections in an image. Microsoft’s COCO [36] data set was fine-tuned to create a domain-specific model capable of identifying individual fibers. To save annotation time, the model is split into three parts, detection, classification, and measurement, that were used in tandem to input an RGB image and output an array of ‘good’ fibers along with pixel-accurate mask areas for each one.

1. **Detection:** A MaskRCNN [37] was trained on manually annotated bounding box data. The detection network data set contained 70 images: 44 used for training and 26 used for validation. Basic data augmentation was used to extend the data set during training. The model was trained for 10 epochs and outputs an array of bounding boxes in the format [x, y, w, h].
2. **Classification:** A combination of Xception [38] and dropout [39] with global max pooling was used to create a classifier network to distinguish between good and bad fibers. A data set of 594 good fibers and 2658 bad fibers was annotated by hand, and data augmentation was used to extend the data set. The classification network was trained for 50 epochs.
3. **Measure:** A MaskRCNN was trained on 200 out of 594 good fibers to output accurate pixel areas. The measure network was trained for 30 epochs.

## 4. Results

This method was designed to detect quantitative differences between different lines of cotton fiber. High-resolution images of individual cotton fibers were acquired and had the lumen area, lumen perimeter, total area, fiber area, fiber perimeter, and circularity measured. The total area had the lumen area subtracted to obtain the true fiber area. These measurements were then used to calculate θ, which is the degree of cell wall thickening, which is calculated by (θ = 4π(Fiber True Area)/(Fiber Perimeter^2^)) [33]. Fiber fineness was determined by calculating the product of the Lumen Area and the density of cotton fibers (1.52 g/cm^3^) [33]. Standard fineness was the final measurement, which is calculated by multiplying fiber fineness by 0.577 then dividing that by θ [33]. All individual measurements were compiled based on their fiber line, creating an average measurement meant to be representative of the line as a whole. Statistical analysis was conducted to ensure that statistically significant differences in measurements were detectable. Each line had around 100 fibers imaged and analyzed to produce the average measurements. An independently analyzed data set from Cotton Incorporated using the same cotton lines was obtained. The data set was used to see if results aligned with a preestablished baseline. The data set only analyzed eight fibers per line, so the data will not fully align due to the higher variability. After the initial analysis was conducted, a histogram was generated to identify outlying data, which removed significantly outlying data from the data set. As fibers were manually selected for measurement analysis, human error could mistakenly include damaged or improperly embedded fibers. This lowered the variability of the measurements without much change to the average values. Algorithmic binning of fibers would remove the need for this process, as a standard will be consistently met.

Table 2 shows three Tukey–Kramer HSD tests for three different properties of cotton fibers. There is no overall grouping between any of the lines. Some lines are more similar to each other than others, but this is just due to similarity between lines, which is inevitable when using multiple samples. The various connecting letter reports show that there is no consistent grouping between lines across measurements. There are somewhat correlated groupings such as with lumen area and lumen perimeter, but these measurements are naturally related to each other, so these measurement groupings are expected.

Measurements each had an all-pairs Tukey–Kramer HSD test conducted to detect statistically significant divergences between the various cotton lines. All measurements had at least three significant groupings among the five lines. The inherent characteristics of cotton fibers resulted in significant variation in measurements among all the lines studied. Surprisingly, the measurements from one line exhibited greater similarity to measurements from other lines than to its own line. Consequently, the data displayed high standard deviations, indicating the substantial variability. Fortunately, the extensive range of measurements conducted demonstrated that no two lines could be grouped together consistently across all the measurements taken based on a Tukey–Kramer HSD test (Table 2). An automated approach for fiber identification and measurement was evaluated. The model had an 84% classification accuracy on the validation data set. Large quantities of analyzed fibers and objective fiber binning would reduce the overlap between fiber line phenotypes.

At least 60 fibers were measured from at least 2 cross-sections for each reference line. Table 3a highlights the differences between the embedding methods for London Resin (LR) embedding and Polymethacrylate (PMMA) embedding. The comparison results ensured that any future LR White results would not have a strong bias in data collection and did not introduce contamination for the measurements. PMMA used a method that required a long curing time that used ultraviolet light in order to polymerize. LR White allows for a quicker polymerization time, and avoiding the use of ultraviolet light allows this technique to be used on more sensitive material. Table 3b shows five unique cotton lines that display different measurements that separate out independently from each other. Each replication verified the embedding technique as not introducing bias into any measurements. For example, the lines Beta and Delta are considerably different, with Beta having a 23% larger lumen area than Delta (Beta:14.83 vs. Delta:12.02) but being within 1% of the fiber fineness (Beta: 241.84 vs. Delta: 244.31). Such different measurements can be found throughout Table 3b, further showing how little bias is introduced via LR White embedding.

Table 3a,b shows that there is a pattern when comparing measurements of the same cotton line. All of the differences in measurements from the LR White lines to the PMMA lines are scaled at a similar rate, as opposed to the different cotton lines seen in Table 3b, which have differently scaled measurement differences for the various lines.

## 5. Discussion

The objective of this study was to simplify the embedding technique for improved analysis of individual cotton fibers. Previous methods utilized PMMA, which is a more intensive method. The methods involving PMMA require the use of UV light and more reactive chemicals, such as benzoyl peroxide. Additionally, these methods require a longer time for polymerization, as PMMA takes around 150 min, while LR polymerization takes less than 20 min. Drying of cross-sections from PMMA can take anywhere from overnight to several days, depending on the embedding thickness. In contrast, the currently proposed method allows the cross-sections to be imaged within 5 min since we are using charged slides, which helps them stick quickly and dry rapidly. Initially, paraffin was employed as the embedding material, and scanning electron microscopy (SEM) was utilized to capture images, followed by ImageJ for image analysis. This method, however, was found to be time-consuming and produced highly variable results with low fiber density per image (Figure 8). Paraffin is a commonly used and cost-effective agent for embedding mammalian tissue due to its simplicity, affordability, and rapid application. However, paraffin embedding can obscure the materials of interest under the microscope and necessitates a deparaffinization process to selectively retain the target material. Furthermore, paraffin embedding lacks the ability to control the orientation of fibers during the sectioning process, often resulting in sections containing horizontally or obliquely arranged fibers (Figure 9A,B).

In this study, we sought to overcome the limitations of traditional embedding methods by exploring the potential of London Resin White (LR White), an acrylic resin that offers numerous polymerization options and exhibits stability at room temperature. LR White resin has been demonstrated to preserve the structural integrity and chemical composition of various biological specimens, making it a promising embedding medium for cotton fibers. The use of N,N-dimethyl-p-toluidine as a chemical accelerator expedited the polymerization process of LR resin, resulting in the preservation of fiber structure within 5–10 min. The resulting embedding medium exhibited a smooth surface after cutting, allowing for high-quality imaging using light microscopy. Both the resin and accelerator used in this study were deemed safe for human use.

LR resin offers superior fiber alignment, resulting in a higher proportion of fibers being optimally oriented (Figure 4A,C). By adopting LR White resin for embedding cotton fibers, we were able to achieve precise control over fiber orientation, improving the accuracy and consistency of fiber measurements. LR White epoxy resin provides a rapid and efficient method for polymerizing biological materials, with several approaches available depending on the material being embedded. Initially, heat-curing was employed but was ultimately deemed unsuitable due to concerns regarding its impact on fiber quality and time efficiency. Instead, the cold-cure method was utilized, incorporating an accelerator to expedite the polymerization time. However, an exothermic reaction can occur during polymerization, necessitating careful experimentation to determine the optimal accelerator concentration that achieves the fastest polymerization rate with minimal heat generation. The use of a thin tube for shaping fibers resulted in significant heat release due to the high surface area-to-volume ratio. Nonetheless, the initial polymerization step ensured the preservation of the fiber shape within the resin, preventing subsequent interference. These results highlight the advantages of using LR White resin for embedding fibrous materials and provide insights for further optimizing polymerization conditions.

Mature fibers are obtained by letting cotton fibers develop from flower to naturally opened cotton boll without outside assistance. Cotton fibers are then allowed to dry without the addition of excessive heat, as this will affect fiber qualities and thus cause a skew in the measurements. The embedding process should result in a pill-sized sample of hardened resin. Be sure to allow the resin to fully polymerize. If not fully polymerized, the sectioning will be problematic and will likely result in poor fiber images in the end (Figure 4B). To verify complete polymerization, the hardness of the resin can be assessed during polymerization by lightly squeezing the gel capsule. Over-polymerization can occur as well, where the resin is very brittle and hard to cut, and this may also result in complications during sectioning. Both issues are related to the concentration of accelerator. Accelerator concentration is the most essential component of embedding, as accelerator-based polymerization is an exothermic reaction. Too much accelerator will release a large amount of heat [40,41] as well as squeeze any of the cotton fiber, both of which will affect fiber properties. Figure 4B represents misformed fibers resulting in excess use of accelerator. Under-polymerization will result in improper sections that will either be impossible to extract or image. Properly polymerized resin should be able to be cut by a straight razor with light force, while not leaving any resin residues after being touched, which is a sign of the resin not being fully polymerized.

After embedding, the fibers were sliced into five-micrometer sections using a glass microtome transferred onto an adhesive microscope slide, and embedding success was determined based on the images obtained from the Olympus LEXT Optical Profiler light microscope. Typical embedding success resulted in 40–60 fibers per section. The glass blades provide ultra-thin sections because they are handmade and sharper than steel blades. Steel blades are unsuitable for achieving a continuously sharp edge due to the crystalline structure of metals [42]. To prevent any disruption to the crystalline structure of the fibers during the embedding process, mature cotton fibers were collected and air-dried at ambient temperature. The current study aims to examine various fiber physical parameters, which is achieved at a 100× resolution. However, the probability of differentiating cell wall layers is minimal at 100× resolution, and this is beyond the scope of this study.

Scanning Electron Microscopy (SEM) was originally used to accurately isolate and measure cotton fibers; however, this approach was time-consuming and costly. In place of SEM imaging, light-based microscopic imaging was utilized. Subsequently, the fibers were manually measured using Adobe Photoshop, which allowed for control over multiple measurements within an image. Despite the method introducing human error, as damaged or obscured fibers had to be excluded, it still produced results similar to an independently analyzed average of the cotton line. Imaging was time-consuming and expensive for SEM, so only healthy fibers were prioritized for imaging, potentially introducing bias into the data set. In contrast, light microscopy can capture larger amounts of fiber simultaneously, allowing a wider variety of fibers to be analyzed. The application of a machine learning approach for fiber phenotyping was explored with positive results. Further adaptation of these models will ultimately allow large numbers of fibers and samples to be rapidly phenotyped and facilitate the adoption of this technique for routine fiber screening. These findings have important implications for the analysis of fibrous materials and provide insights for future studies aiming to optimize cotton fiber phenotyping.

## Figures and Tables

**Table 1:** SEM vs. light fiber measurements.

	Light Microscope					Scanning Electron Microscope				
Reference Cotton Line	Outer Perimeter (μm)	Outer True Area (μm^2^)	θ	Fiber Fineness μm×g/cm	Maturity Ratio (μm^2^)	Outer Perimeter (μm)	Outer True Area (μm^2^)	θ	Fiber Fineness μm×g/cm	Maturity Ratio (μm^2^)
2888	48.07	102.58	0.56	155.92	0.97	48.73	104.05	0.55	158.16	0.95
3159	60.40	132.96	0.48	202.10	0.83	64.78	143.83	0.43	218.62	0.75
3169	60.86	119.36	0.42	181.43	0.72	62.65	119.04	0.38	180.94	0.66
3212	55.70	131.87	0.55	200.44	0.95	57.95	144.98	0.54	220.37	0.94
3214	51.80	106.37	0.51	161.68	0.89	53.92	110.59	0.48	168.10	0.83

**Table 2:** Tukey–Kramer HSD test for lumen perimeter, outer area, and θ.

Lumen Perimeter (μm)							Outer True Area					θ					
Line						Mean	Line				Mean	Line					Mean
3169	A					37.24	Delta	A			160.73	3159	A				0.93
Gamma	A					36.12	Beta	A			159.11	2888	A				0.93
Beta	A	B				33.91	Gamma	A	B		136.72	3212	A				0.93
Alpha	A	B	C			32.62	3159		B		132.96	Delta	A				0.93
3159	A	B	C	D		31.52	3212		B		131.87	3214	A	B			0.92
Theta		B	C	D	E	28.02	3169		B	C	119.36	Beta	A	B	C		0.91
3212			C	D	E	27.72	Alpha		B	C	115.49	Gamma		B	C	D	0.90
Delta			C	D	E	26.94	3214			C	106.37	3169		B	C	D	0.90
3214				D	E	26.31	Theta			C	104.92	Theta			C	D	0.89
2888					E	24.42	2888			C	102.58	Alpha				D	0.89

**Table 3:** (**a**) Comparison of mean values for reference cotton lines using LR and PMMA. (**b**) Mean values for reference cotton lines using LR.

(**a**)										
	**London Resin White (LR)**					**Polymethyl Methacrylate (PMMA)**				
**Reference Cotton Line**	**Outer Perimeter (μm)**	**Outer True Area (μm^2^)**	**θ**	**Fiber Fineness μm×** **g/cm**	**Maturity Ratio (μm^2^)**	**Outer Perimeter (μm)**	**Outer True Area (μm^2^)**	**θ**	**Fiber Fineness μm×** **g/cm**	**Maturity Ratio (μm^2^)**
2888	48.07	102.58	0.56	155.92	0.97	47.20	101.50	0.58	154.28	1.00
3159	60.40	132.96	0.48	202.10	0.83	57.80	125.00	0.49	190.00	0.85
3169	60.86	119.36	0.42	181.43	0.72	54.00	114.90	0.51	174.65	0.89
3212	55.70	131.87	0.55	200.44	0.95	48.40	94.40	0.61	143.49	0.90
3214	51.80	106.37	0.51	161.68	0.89	47.10	103.20	0.52	156.86	1.03
(**b**)										
**Line**	**Lumen Area (μm^2^)**	**Lumen Perimeter (μm)**	**Lumen Circularity**	**Outer Perimeter (μm)**	**Outer True Area (μm^2^)**	**Outer Circularity (μm)**	**θ**	**Fiber Fineness μm×** **g/cm^3^**	**Maturity Ratio** **(μm^2^)**	**Standard Fineness**
Alpha	13.59	32.62	0.17	55.45	115.49	0.53	0.89	175.54	1.54	113.20
Beta	14.83	33.91	0.19	64.22	159.11	0.54	0.91	241.84	1.58	152.55
Delta	12.02	26.94	0.24	61.33	160.73	0.58	0.93	244.31	1.61	151.51
Gamma	15.04	36.12	0.17	62.02	136.73	0.51	0.90	207.82	1.56	133.10
Theta	11.80	28.02	0.23	52.23	104.92	0.54	0.89	159.48	1.54	102.37

## Data Availability

The data sets and developed models from this procedure are publicly available on GitHub https://github.com/RifeLab/cotton-lumen (accessed on 20 August 2023).
